# Post-traumatic Stress Disorder and Suicidal Behaviors in American Adolescents: Analysis of 159,500 Psychiatric Hospitalizations

**DOI:** 10.7759/cureus.8017

**Published:** 2020-05-07

**Authors:** Noha Eskander, Ramu Vadukapuram, Shaheer Zahid, Sahar Ashraf, Rikinkumar S Patel

**Affiliations:** 1 Psychiatry, Ain Shams University Hospital, Cairo, EGY; 2 Psychiatry, State University of New York Upstate Medical University, Syracuse, USA; 3 Psychiatry, Saint James School of Medicine, Park Ridge, USA; 4 Psychiatry, Mayhill Hospital, Denton, USA; 5 Psychiatry, Griffin Memorial Hospital, Norman, USA

**Keywords:** ptsd, child and adolescent psychiatry, suicide risk, posttraumatic stress

## Abstract

Objectives

Our main goal is to understand the demographics and psychiatric comorbidities and to evaluate the risk of suicidal behaviors in post-traumatic stress disorder (PTSD) adolescents.

Methods

We included 159,500 adolescents (age, 12 to 18 years) with a primary psychiatric diagnosis from the Nationwide Inpatient Sample from January to December 2014 and grouped them by a diagnosis of PTSD (N = 21,230 [13.3%]). A logistic regression model was used to measure the odds ratio (OR) for suicidal behaviors in PTSD versus non-PTSD cohorts.

Results

A higher proportion of PTSD adolescents were females (75.7%) and whites (63.6%). The most prevalent psychiatric comorbidities in PTSD inpatients (vs. non-PTSD) were anxiety disorders (100% vs. 31.9%) and mood disorders (89.4% vs. 84.7%). About 48.7% of PTSD inpatients had suicidal behaviors and had a higher risk (OR 1.23; 95% CI: 1.19-1.26; P < 0.001) compared to that seen in 43.6% of the non-PTSD cohort.

Conclusions

Diagnosis of PTSD is prevalent in adolescents, especially females and whites, with anxiety and mood disorders being the most prevalent comorbidities. There exists a significant association between PTSD and suicidal behaviors, with an increased risk of 23% in adolescents.

## Introduction

Post-traumatic stress disorder (PTSD) is a psychiatric illness that develops after an individual is exposed to traumatic life events. The prevalence of trauma among high-risk children was about 15% to 43% in girls and 14% to 43% in boys who were subjected to at least one traumatic event in a lifetime. Of these, at least 3% to 15% of girls and 1% to 6% of boys develop PTSD [1]. Children show less distress due to which the prevalence of PTSD may be lower in children than adults. The risk of PTSD is seen in a higher proportion of white females and adolescents (12 to 14 years) those from lower socioeconomic status, and rural residents [2]. Other risk factors include negative coping behaviors, lack of support system, and chronic childhood trauma [3].

PTSD leads to changes in brain anatomy and neurophysiology. The size of the hippocampus is inversely related to the severity of PTSD [4]. The amygdala, which is responsible for emotion regulation, appears to overreact, and the medial prefrontal cortex, which is responsible for the inhibition of emotional over-reactivity of the amygdala, appears to be less responsive in PTSD patients [5].

Adolescents with PTSD have a higher suicidal risk due to interpersonal assault, rape, and natural disasters [6,7]. Adolescents with a history of trauma and PTSD diagnosis have increased risk of self-harm, suicidal ideation, and suicide attempt compared with adolescents with trauma history only [7]. Adolescents with a diagnosis of PTSD have 15 times increased suicidal risk compared with adolescents without PTSD, possibly due to worsening symptoms such as intrusive thoughts, irritability, and anger with poor impulse control [7,8]. Also, individuals with PTSD who struggle with feelings of hopelessness, defeat, and entrapment are also at high risk of suicide [9].

We used the inpatient data from the United States hospitals to understand the differences in demographics and psychiatric comorbidities in adolescents with PTSD versus other psychiatric inpatients. Next, we evaluated the risk of suicidal behaviors in adolescents diagnosed with PTSD.

## Materials and methods

Data source

We conducted a cross-sectional data analysis using the Nationwide Inpatient Sample (NIS) from January to December 2014 [10]. These data provide inpatient records from about 4,400 community-based hospitals across 44 states in the United States [10]. Diagnostic information in the NIS is detected using the International Classification of Diseases, Ninth Revision (ICD-9) codes and the Clinical Classifications Software (CCS) codes [10]. As we used a de-identified NIS data in our study, we do not require approval from the Institutional Review Board.

Inclusion criteria and outcome variables

We included 159,500 adolescent inpatients (age 12 to 18 years) with a primary discharge diagnosis of psychiatric conditions. We then compared the groups with a primary diagnosis of PTSD using the ICD-9 diagnostic code 309.81 (N = 21,230) versus a group with other psychiatric diagnoses (non-PTSD cohort, N = 138,270). And, the co-diagnosis of suicide and the intentional self-inflicted injury was identified using the CCS code 662.

Demographic characteristics included age, sex (male or female), and race (white, black, Hispanic, and others) [11]. The comorbidities included in our study are attention-deficit/hyperactivity disorder (ADHD)/conduct/behavioral disorders (CCS code: 652), anxiety disorders (CCS code: 651), mood disorders (CCS code: 657), schizophrenia and other psychotic disorders (CCS code: 659), alcohol abuse (CCS code: 660), and substance abuse (CCS code: 661).

Statistical analysis

We used descriptive statistics and chi-square test to evaluate the differences in demographic and psychiatric comorbidities, and suicidal behavior in PTSD vs. non-PTSD cohorts. Logistic regression analysis was used to measure the odds ratio (OR) of suicidal behavior in PTSD inpatients after comparing it with the non-PTSD cohort. A P-value of <0.01 was considered for statistical significance, and data analyses were performed using the SPSS Version 26 (IBM Corp., Armonk, NY, USA).

## Results

We analyzed data from 159,500 adolescent (mean age: 15 years) psychiatric inpatients, and 13.3% had a primary discharge diagnosis of PTSD. A higher proportion of the PTSD inpatients were females (75.7%) compared with the non-PTSD cohort (59.3%). PTSD inpatients were majorly whites (63.6%) followed by blacks (16.6%) and Hispanics (12.5%), but there was statistically no significant difference in racial distribution when compared with non-PTSD (P = 0.036).

The most prevalent psychiatric comorbidities in PTSD inpatients (vs. non-PTSD) were anxiety disorders (100% vs. 31.9%), mood disorders (89.4% vs. 84.7%), and ADHD/conduct disorder/disruptive behavioral disorders (36.5% vs. 33.1%). Schizophrenia and other psychotic disorders, and substance abuse were seen in a lower proportion of PTSD inpatients compared with the non-PTSD cohort, as shown in Table 1.

**Table 1:** Demographic predictors and risk of suicidal behaviors in post-traumatic stress disorder N, number of patients; ADHD, attention-deficit/hyperactivity disorder

Variable	Post-traumatic stress disorder		P-value
	No	Yes	
Total. N	138,270	21,230	-
Mean age, years	15.33	15.16	<0.001
Sex, %			
Male	40.7	24.3	<0.001
Female	59.3	75.7	
Race, %			
White	62.5	63.6	0.036
Black	16.6	16.6	
Hispanic	13.6	12.5	
Other	7.4	7.3	
Comorbidities, %			
Anxiety disorders	31.9	100.0	<0.001
ADHD/conduct/behavioral disorder	33.1	36.5	<0.001
Mood disorders	84.7	89.4	<0.001
Schizophrenia and other psychotic disorders	8.4	6.4	<0.001
Alcohol abuse	7.1	7.2	0.574
Substance abuse	22.4	20.9	<0.001

Around 48.7% of PTSD inpatients had suicidal behaviors, which was significantly higher than that seen in 43.6% non-PTSD cohort (P < 0.001). PTSD inpatients had 1.2 times higher odds for suicidal behaviors (95% CI: 1.19-1.26; P < 0.001) compared with the non-PTSD cohort, as shown in Table 2.

**Table 2:** Suicidal behaviors in adolescent inpatients N, number of patients

Suicidal behavior	Post-traumatic stress disorder				Logistic regression analysis		
	No		Yes		Odds ratio	95% CI	P-value
	N	%	N	%			
Non-suicidal	77,935	56.4	10,890	51.3	Reference		
Suicidal	60,335	43.6	10,340	48.7	1.23	1.19 – 1.26	<0.001

Four-fifths (80%) of PTSD inpatients with suicidal behaviors were females and 65.9% were whites, as shown in Table 3.

**Table 3:** Demographic distribution of post-traumatic stress disorder inpatients by suicidal behavior N, number of patients

Variable	Suicidal behaviors	
	No	Yes
Total, N	10,890	10,340
Mean age, years	15.13	15.20
Sex, %		
Male	28.5	20.0
Female	71.5	80.0
Race, %		
White	61.4	65.9
Black	18.2	14.9
Hispanic	12.8	12.0
Other	7.6	7.1

## Discussion

In our data analysis of 159,500 adolescents, we found a statistically significant association between PTSD and suicidal behaviors. After comparing with other psychiatric adolescent inpatients, a diagnosis of PTSD increased the risk of suicidality by 23%.

According to a meta-analysis (N = 3,563), PTSD was prevalent in 15.9% of adolescents who were exposed to trauma at least once in their lifetime [12]. This was also seen in our inpatient population with 21,230 (13.3%) patients diagnosed with PTSD. Females are two to three times higher risk to develop PTSD as they are more likely to be exposed to traumatic events such as childhood sexual assault and rape at a younger age than males [13]. Females use the tend-befriend response to cope with stressful situations, whereas males use the fight-flight response in facing similar traumatic events [14,15]. As males use problem-focused coping strategies comparable with emotion-focused strategies in females, females tend to report more anxiety and depression symptoms and are more likely to present with symptoms that meet the diagnostic criteria for PTSD compared with males [14,15]. Females appear to have a more sensitized hypothalamic-pituitary-adrenal (HPA) axis, whereas males have a sensitized physiological hyperarousal system. These psychobiological differences can potentially explain the difference in gender rates for PTSD [14]. These are the possible reasons why we found three-fourths of the PTSD inpatients in our study to be females.

Whites are more likely to be exposed to and also report any trauma-related events than other races [16]. Blacks are more subjected to events such as childhood maltreatment and domestic abuse [16]. But blacks are less likely to report traumatic events due to limited healthcare coverage. Also, they respond more physically to stressful events than psychologically, which leads to a bias in PTSD diagnosis [17]. There was a statistically non-significant difference in terms of racial distribution between PTSD and other psychiatric inpatients in our study. Yet, three-fifths of the PTSD inpatients were whites, which do support current literature [16,17].

Lower serotonin levels in the brain are seen in PTSD, which leads to anxiety, depression, and increased suicidal risk [18]. Around 60% of genetic similarities between PTSD, and generalized anxiety disorders, obsessive-compulsive disorder, and panic disorders exist due to serotonin gene polymorphism [19,20]. Half of the adolescents suffering from PTSD may also suffer from major depression as both illnesses are associated with a dysregulation of the HPA axis [21]. Thus, in our PTSD inpatients, anxiety disorders and mood disorders were the most prevalent psychiatric comorbidities.

Past studies have found that suicide risk is higher in PTSD adolescents and is mainly caused by genetic factors or/and environmental factors [22]. Traumatic grief, childhood abuse, and peer suicide are among the most common risk factors followed by psychobiological factors such as negative coping mechanisms with the trauma, worrying, impulsivity, alcohol, and substance abuse that increase the suicidality in adolescents with PTSD [22,23]. Negative self-appraisal and feeling of entrapment and defeat are the core findings in adolescents with suicidal ideation and PTSD [24]. Individuals with suicidal behaviors due to PTSD have shown an increase in metabotropic glutamate receptor type 5 (mGluR5) availability in the frontal-limbic region of the brain, whereas this was not seen in depressed individuals with suicidal thoughts [25].

A study in a community sample (N = 1,698) of urban American young adults found a higher suicide rate (relative risk: 2.7; 95% CI: 1.3-5.5; P <0.01) and was adjusted for comorbid psychiatric disorders [7]. But an important limitation was the small sample size, and most participants were black males living in the urban metropolitan areas [7]. In our study, we used the national population and found that PTSD increases the risk of suicide by 1.2 times in adolescents compared with other psychiatric inpatients. Also, 80% of the suicidal PTSD patients were females, which could be possibly related to higher sexual assault seen in young females and may potentially increase suicide rates in females [26].

An important limitation of our study include is that the NIS data lack individual-level clinical information and cross-sectional nature of the study that prevents from confirming the causal relationship between suicidal behavior in PTSD adolescents. But, using these national data, we were able to confirm a population-based suicidal association in PTSD adolescents after controlling for possible confounders.

## Conclusions

Diagnosis of PTSD is prevalent in adolescents, especially females and whites, with anxiety and mood disorders being the most prevalent comorbidities. There exists a significant association between PTSD and suicidal behaviors, with an increased risk of 23% in adolescents. Current PTSD checklist needs modifications to address the gaps in terms of comorbid anxiety and depression and cover demographic differences in response to trauma. More research is required to better understand the genetic differences and neurobiochemical changes in adolescents with PTSD and suicidal behaviors.
